# “Rise amid the global uncertainty and chaos” socio-environmental threats risk towards quality of life, mental health and psychological well-being of young adults in Malaysia

**DOI:** 10.3389/frcha.2026.1854310

**Published:** 2026-07-30

**Authors:** Siti Khadijah Zainal Badri, Khalif Farhan Abdul Mutallib

**Affiliations:** School of Psychology, University of Nottingham Malaysia Campus, Semenyih, Malaysia

**Keywords:** threat and uncertainty, mental health, quality of life, psychological well-being, young adult

## Abstract

**Introduction:**

This study examines the impact of socio-environmental threats on various aspects of young adults' well-being in Malaysia. It focuses on understanding the odds posed by different threats in relation to broader global challenges to this population's quality of life, mental health, and psychological well-being.

**Methods:**

A cross-sectional quantitative design with a sample of 508 young adults was used in this study. Data were analysed using logistic regression.

**Results:**

The findings reveal that key threats to well-being, namely economic instability, career uncertainty, and family pressures, significantly impact the quality of life, mental health, and psychological well-being of young adults in Malaysia. Additionally, the threat of war is perceived to increase the odds of poor personal development. Global and environmental threats, along with societal expectations, were associated with greater odds of positive behaviour. Interestingly, concerns about unemployment were associated with stronger social bonding, suggesting varied responses to different threats.

**Discussion:**

This study highlights the socio-environmental factors influencing young adults' well-being in Malaysia through an explorative lens. Future research could delve deeper into the specific sentiments of young adults and explore these threats and uncertainties from more focused perspectives.

## Introduction

The evolution of global and local socio-environmental challenges has heightened society's concern today. Undeniably, the world is in a phase of evolving, escalating crises across economic, political, social, and environmental spheres that seem to threaten the future of the human population. Each year, more action calls are made for scientists, educators, and stakeholders to respond to the “world-in-crisis” that befalls our humanity. In Malaysia, one of the crises the country is facing is the rise of a “silent” mental health epidemic among young people. Situated in Southeast Asia, Malaysia is a relatively small country that thrives on diversity and collective excellence. Commonly reported risk factors for mental health in the region include academic stress, relationship issues, socioeconomic status, and cultural richness, according to Kok and Low's (32) review. Some cultural aspects have driven mental health to be a taboo topic, with different ethnicities having varied conceptual beliefs associated with mental health struggles (1, 2).

Studies on the pandemic (3) and climate (4) have sparked more discourse on the influence of environmental challenges on young Malaysians' well-being. Additionally, economic volatility is of greater concern to this population than before (5). However, there is very little, if any, comprehensive study that examines different types of social and environmental threats in a single study, especially to understand how they affect young adult well-being. This paper uses Bronfenbrenner's (6) Social-Ecological Model of cultural psychology and General Strain Theory (7) to examine the influence of these threats on young adults. It mainly argues that young adult well-being can be a byproduct of broader exposure to or interaction with current global challenges across different system levels, drawing on Bronfenbrenner's (6) theory and threat perception from General Strain Theory (7) in the surrounding socio-environmental landscape.

It seeks to supplement the reductionist view of well-being research, which often focuses on biological, chemical, and neurological components of the human body. This study draws on an interdisciplinary ecological perspective on the individual well-being outcome within different system experiences and threat reactions, given the mixed challenges young adults face across various parts of the system in Malaysia. Using Bronfenbrenner's ecological systems theory as a conceptual framework, this study situates the individual student within a dynamic network of environmental influences, emphasising the role of macro-level global disruptions, including political, economic, and health-related uncertainties, in shaping students' perceptions of risk and its effects on their different components of well-being in this study (i.e., Quality of life, psychological wellbeing and mental health).

## Context of the study

In Malaysia, there has been a notable rise in mental health issues among young adults, with significant concerns regarding the unmet psychological needs reported for the population (33). Mental health stability for the young generation is seen as a crucial national agenda to ensure the nation's prosperity, focusing on a quality workforce and social well-being. Studies have suggested that poorer mental health during early adulthood is strongly linked to an increased risk of substance abuse, violent behaviours, and other problematic relationships as well as conduct (8). Besides, poorer mental health links to more suicide problems, with the 2022 National Health and Morbidity Survey (NHMS) revealing alarming rates of suicidal ideation (13.1%) and attempted suicide (9.5%), with higher prevalence among girls (34).

A safe and supportive environment plays a vital role in adolescent development, significantly influencing self-confidence and long-term success. Conversely, exposure to unsafe or threatening environments can provoke psychological responses such as fear and anxiety, which may adversely affect adolescents' mental health. This is highlighted by General Strain Theory (7). Despite the theory focusing on how threat predisposes anger and criminal behaviour, it also highlights the mental health consequences of surrounding threats. Given that adolescence is a critical developmental stage, environmental stressors, whether social, political, or environmental, can have long-lasting consequences. This is somehow evident in global studies on climate change linked with negative emotions (sad, anxious, angry, powerless) and disrupted daily functioning due to excessive worries (9) that parallel many other studies (10–12).

In recent years, global crises such as the COVID-19 pandemic, climate change, and geopolitical tensions have intensified psychological stress (9, 13, 14). The COVID-19 pandemic, for example, has left deep psychological scars, exacerbating issues like loneliness, depression, and suicidal tendencies (13, 15). Meanwhile, climate change, driven by factors such as deforestation and overconsumption, has increased the frequency of extreme weather events, including floods, creating new anxieties about the future [e.g., (16, 17)]. Ongoing geopolitical conflicts, such as war and genocide, have further contributed to global-scale fear and uncertainty, especially among vulnerable populations like young adults (14, 18).

Despite the growing awareness of mental health challenges in the context of threats, limited research has explored how these varying threats contribute to psychological distress among young people in the Malaysian region. Comprehensive studies are often limited, as most studies rely on single threat scenarios such as climate change and covid-19 (4, 19), among others. To date, there is a lack of studies examining a comprehensive list of scenarios associated with social-environmental threats or stressors. Even more limited is a study that provides some evidence on how such existential and evolving threats relate to the well-being of young people in Malaysia. This paper aims to fill in those gaps by providing evidence on how emerging and evolving global threats, pandemics, climate change, natural disasters, and war are linked to young adult psychological well-being in the Malaysian region.

## Threat as negative emotional response

Perceived threats, whether social, environmental, or existential, elicit a certain degree of emotional responses. This is evident in global studies on climate change by Hickman et al. (9), which link to adverse emotional reactions such as sadness, anxiety, anger, and powerlessness. According to General Strain Theory (7), stress generates negative emotions, which may trigger maladaptive coping in response to perceived danger. This aligns with the general threat hypothesis, which posits that individuals perceive a situation as threatening to their survival, well-being, or identity, and that the brain activates emotional and physiological responses, such as fear, anxiety, and helplessness, as described by the cognitive appraisal theory of emotion (20). While these reactions are adaptive in the short term, their prolonged occurrence can lead to maladaptive emotional states and contribute to long-term psychological distress.

The emotional impact of perceived threats is primarily shaped by cognitive appraisal, which involves assessing severity and the capacity to cope with it (20). Clinically, extreme, high-intensity and repeated threats within the immediate environment have been associated with increased risk of mental health problems (21). Flanelly et al. (35) also highlighted that beliefs about the world influence mental health through the assessment of harm, which involves a series of brain processes mainly occurring in the basal ganglia, limbic system, and prefrontal cortex. Extreme events such as bullying or familial conflict often provoke emotions like fear and shame. In contrast, broader threats, such as the COVID-19 pandemic or climate change, may induce feelings of helplessness, anxiety, and existential dread (9), particularly when perceived as uncontrollable.

## Young adult psychological state and socio-environmental challenge

The Bronfenbrenner ecological theory (6) perspective is utilised in this study to understand how broader ecological systems and young adult' varying experiences of emotional responses to threat and subsequently their well-being. Although this model is often applied in childhood development, it has also been used to understand individual responses to challenges in adulthood [e.g., (22)].

According to Perron (22), young adults will experience a significant shift and form their own perception as they move between different levels of systems. Microsystem-level concerns involve immediate environmental interactions, such as family, peers, and school, which are supportive. This system involves extensive personal experience and exchange, from an intimate to a closed-space network. An exosystem, on the other hand, encompasses broader societal structures, such as the media and community institutions. It is an expanded system in which individuals will begin to understand mixed views and the complexity of community concerns. At the macrosystem level, individuals begin to comprehend many general, often uncontrollable subjects that may or may not affect them, including global issues, cultural norms, and political systems.

This paper argues that interactions across different system levels shape how young people view and respond to threats at both foundational and expanded levels. For example, continuous exposure to distressing media coverage of prolonged economic volatility may exacerbate feelings of fear and anxiety, thereby affecting their psychological well-being. Besides, young adults' views on the struggles to address escalating global crises can either alleviate or amplify their worry about future prosperity and peace.

From a threat perspective (20), it is understandable that both socio- and environmental stressors, particularly those perceived as uncontrollable or unpredictable, lead to heightened emotional states. The perception of a lack of resources and ability to fully process and manage such stressors can result in generalised anxiety symptoms as reported in many research studies discussing threats (9, 13, 14). In Malaysia, a small number of studies have established a negative link between harmful or extreme threat events such as flooding, extreme heat, economic concern and climate anxiety with psychological distress. A brief study done by Othman et al. (23) on flood victims found some link to emotional anxiety and fear-related emotions. Additionally, a study by Lateh et al. (24) found that affected landslide victims in Malaysia reported signs of distress and post stress.

Understandably, emotional responses to perceived threats among young people are shaped by both cognitive perception processes and broader environmental interaction factors. Understanding its effects on well-being requires comprehensive, integrated knowledge of how complex environmental interactions and perceptions shape threats. As such, this paper draws on knowledge from both angles to understand young adults’ perceptions of surrounding socio-environmental threats in the local and global contexts. It aims to understand how existing threat conditions are perceived and how they relate to the well-being of this population, across different mental health conditions and aspects of quality of life.

## Methodology

### Design

This study utilised a cross-sectional quantitative design and a self-report questionnaire. Data were collected online from young adults aged 18–30 years. All samples are university students aged 18–30, in line with the Malaysia Youth Societies and Youth Development (Amendment) Act 2019.

### Samples and distribution strategy

A total of 508 university students were recruited for this study. Participants were recruited through online dissemination of the survey link across various social media platforms and university networks. Participation was voluntary and based on the study inclusion criteria. Given the 19 predictor variables examined, the final sample size exceeded commonly recommended guidelines for logistic regression analysis (25) and was considered adequate for the analyses performed. Calls for participation are shared across various social media platforms. All participants were asked for their consent, informed of anonymity, and given the right to withdraw from the survey before answering.

### Instruments development

Threat perception items—The threat and uncertainty items were measured using a customised 19-item scale. All items are developed using a single statement, based on the literature on different types of socio-environmental challenges that might affect young adults' well-being, and are unique to the Malaysian population. To ensure a more comprehensive coverage of contemporary threat conditions, Generative Artificial Intelligence (AI) was used as a supplementary brainstorming tool to explore additional threat scenarios. The AI-generated suggestions were subsequently triangulated with the literature and reviewed by the research team to ensure theoretical relevance and contextual suitability to the Malaysian setting. The final list of items was then refined and pilot-tested using 30 samples to assess readability, clarity, and language appropriateness to the local context before the commencement of the actual study. All items are designed to assess threat perception from an emotional perspective and obtain an accurate answer. The complete list of items is sent for pilot testing to assess readability and language expression before the study begins. All statements are rated with “Yes” or “No” to determine whether a particular threat condition was perceived as relevant or threatening by respondents. The focus was on identifying the presence or absence of concern rather than its severity or frequency. Responses were coded into binary categories (0 = No, 1 = Yes), with the higher-risk category used as the reference group in the analysis.

Well-being—Young adults' well-being in this study is measured by three variables: psychological well-being, quality of life, and mental health. *Psychological well-being* was measured using the 18-item Ryff scale. The scale consists of 6 dimensions: autonomy, environmental mastery, life purpose, positive relations, personal growth, and self-acceptance (36). Individuals were required to respond on a 6-point Likert scale to indicate how true each statement was for them. *Quality of life* was measured using the Quality-of-Life Scale (QOLS), discovered by American Psychologist John Flanagan. The 16-item scale measures five domains: physical well-being, relationships with others, social, communities, and civic activities. Respondents rated their quality of life on a 7-point Likert scale across five dimensions (37). Meanwhile, *Mental health* was measured using the DASS-21, which stands for the Depression, Anxiety, and Stress scale. Participants had to respond to a 4-point Likert scale to measure those three negative emotions.

### Data analysis

We performed a logistic regression to investigate the odds of threat to predict the occurrence of high levels of mental health symptoms, as well as poor quality of life, and psychological well-being problems. This approach is widely used in mental health research to identify factors associated with a binary outcome (e.g., presence/absence, agreement/disagreement). Odds ratios (ORs) were used to estimate the strength and direction of associations between threat perceptions and well-being outcomes. Prior to conducting logistic regression, multicollinearity diagnostics were performed among the predictor variables. The Variance Inflation Factor (VIF) values ranged from 1.30 to 1.88, while tolerance values ranged from 0.53 to 0.77, indicating no evidence of serious multicollinearity among the predictors.

## Findings

Table 1 summarises the odds ratio for the predictive role of threats and uncertainty in the quality of life (QOL) of young adults, based on five main components: physical and material wellbeing, relationships, social community and civic engagement, personal development, and recreation, using a sample of 508. In summary, the 19 items were created across different scenarios of possible threats (yes/no), and analysis was performed using logistic regression. Several interesting and important findings regarding the risk of threats to QOL components were identified.

**Table 1:** Odds ratio for the predictive role of threats and uncertainty towards risk of poor^n^ quality of life (QOL) based on five components among young adults in Malaysia.

Threats and Uncertainty Question	QOL physical and material well-being		QOL relationship		QOL social, community and civic		QOL personal development		QOL recreation	
	*p*-value	Exp (*B*)	*p*-value	Exp (*B*)	*p*-value	Exp(*B*)	*p*-value	Exp(*B*)	*p*-value	Exp(*B*)
1. The thought of not having a job after graduation makes me stressed/anxious	.271	1.452	.024	2.222*	.491	1.276	.003	3.217*	.098	1.908
2. Uncertainty about the future makes it difficult for me to plan my career path.	.809	.929	.260	.684	.924	1.031	.569	.807	.224	.641
3. Family expectations add pressure to making financially secure career choices, even if they do not align with my interests.	.147	1.584	.945	1.024	.234	1.485	.936	.969	.607	1.207
4. I sometimes sacrifice my psychological well-being to meet career and financial expectations.	.104	1.608	.039	1.898*	.009	2.185*	.201	1.567	.126	1.660
5. I worry that economic instability will make it difficult to secure a stable career.	.023	2.455*	.058	2.253*	.783	1.125	.282	1.637	.414	1.459
6. The rising cost of living makes me uncertain about achieving financial stability.	.427	1.361	.342	1.476	.031	2.333*	.034	2.523*	.174	1.783
7. Societal expectations make me feel obligated to prioritise financial success over personal fulfilment.	.604	.851	.833	.934	.039	.499*	.436	.746	.097	.543
8. Uncertainty about future economic conditions makes it difficult to save or invest for the long term.	.311	1.401	.594	1.208	.528	1.244	.043	2.160*	.047	2.056*
9. I feel pressure to achieve success quickly due to fears of future financial instability.	.149	.608	.283	.677	.071	.521	.214	.602	.411	.730
10. Financial instability causes me stress and anxiety about my future quality of life	.933	.972	.337	.699	.993	.997	.431	.716	.883	.944
11. Societal expectations about success and financial stability have an impact on my self-esteem and mental health.	.431	.768	.233	.654	.076	.534	.043	.429	.012	.363
12. The pressure to meet family expectations makes me feel overwhelmed and emotionally drained	.198	1.572	.294	1.472	.007	2.642*	.019	2.712*	.002	3.510*
13. I worry that economic instability will limit my ability to maintain a good quality of life.	.328	.697	.073	.484	.620	.832	.026	.361	.192	.578
14. I feel uncertain about starting a family due to concerns about financial and emotional stability.	.519	.805	.846	1.070	.575	.826	.756	1.127	.231	1.548
15. I feel anxious about how nuclear weapons or chemical warfare could create long-term environmental disasters	.933	1.030	.938	1.029	.447	.759	.086	.474	.281	.645
16. Concerns about climate change negatively impact my mental well-being	.807	.924	.420	1.317	.738	1.118	.361	1.436	.200	1.599
17. I feel anxious about how environmental degradation will affect my future quality of life.	.881	.947	.894	.950	.840	.928	.649	1.212	.960	.980
18. The unpredictability of climate-related disasters contributes to my stress and anxiety.	.262	.684	.089	.541	.330	.712	.266	.639	.061	.485
19. The risk of war makes me feel uncertain about my personal safety and well-being.	.804	.922	.942	.976	.112	1.669	.029	2.194*	.232	1.528

n QOL with moderate, low and very low levels.

* significant at *p*-value less than .05.

Foremost, threats related to economic instability and unstable careers were found with increased odds of declining physical and material well-being among the young adults. In detail, the item “*I worry that economic instability will make it difficult to secure a stable career*” was associated with 2.4 times increased odds of poorer physical and material wellbeing [Exp(*B*) = 2.455, *p* = 0.023]. Poorer QOL relationship is found to be associated with three statements of threat. Specifically, the worry associated with the threat of not having a job (*The thought of not having a job after graduation makes me stressed/anxious*) was associated with a 2.2 increased risk [Exp(*B*) = 2.222, *p* = 0.024], “*I sometimes sacrifice my psychological well-being to meet career and financial expectations*” associated with a 1.9 higher odds [Exp(*B*) = 1.898, *p* = .009] as well as “*I worry that economic instability will make it difficult to secure a stable career*” which predicted 2.2 odds of poorer QOL in terms of relationship [Exp(*B*) = 2.253, *p* = .058] albeit borderline.

Regarding QOL social, community, and civic components, the findings identified three risk factors and one protective factor*.* “*I sometimes sacrifice my psychological well-being to meet career and financial expectations*” is associated with 2.1 times odds [Exp(*B*) = 2.185, *p* = 0.009], “*The rising cost of living makes me uncertain about achieving financial stability*” is associated with 2.3 times [Exp(*B*) = 2.333, *p* = 0.031] and “*The pressure to meet family expectations makes me feel overwhelmed and emotionally drained*” is associated with 2.6 odds [Exp(*B*) = 2.642, *p* = .007] of poor QOL on the social, community and civic aspect. Interestingly, we found that pressure associated with expectation “*Societal expectations make me feel obligated to prioritise financial success over personal fulfilment.*” decreases the odds of poor QOL in this domain [Exp(*B*) = 0.499, *p* = .039]. Moving on, QOL personal development is found to be the component with the most significant result. Under the dimension, we found five conditions associated with increased odds of poor QOL personal development, ranging from 2.1 to 3.2 times greater. These are “*The thought of not having a job after graduation makes me stressed/anxious*” [Exp(*B*) = 3.217, *p* = 0.003], “*The rising cost of living makes me uncertain about achieving financial stability*” (*B* = 2.523, *p* = 0.034), “*Uncertainty about future economic conditions makes it difficult to save or invest for the long term*” [Exp(*B*) = 2.160, *p* = 0.043], “*The pressure to meet family expectations makes me feel overwhelmed and emotionally drained*” [Exp(*B*) = 2.712, *p* = 0.019] and “*The risk of war makes me feel uncertain about my personal safety and well-being*” [Exp(*B*) = 2.194, *p* = 0.029]. Lastly, QOL recreation is found to be associated with only two statements of “*Uncertainty about future economic conditions makes it difficult to save or invest for the long term*” and “*The pressure to meet family expectations makes me feel overwhelmed and emotionally drained*” with both predicted 2 [Exp(*B*) = 2.056, *p* = .047] as well as 3.5 [Exp(*B*) = 3.510, *p* = 0.002] occurrences of poorer QOL in future.

The findings on the predictive risk of different threat conditions unique to young adult mental health symptoms, based on moderate to extreme DASS-21 scores, are presented in Table 2 above. Finding reveals only a few significant results towards the increased occurrence of mental health problems. Particularly, uncertainty related to career, “*Uncertainty about the future makes it difficult for me to plan my career path*” (*B* = 2.406, *p* = .009), “*Financial instability causes me stress and anxiety about my future quality of life*” (*B* = 2.517, *p* = 0.13) is associated with increased occurrence of moderate to high level of anxiety symptoms. Besides, “*The pressure to meet family expectations makes me feel overwhelmed and emotionally drained*” item is associated with a five times higher odds of moderate to high levels of depression (*B* = 5.706, *p* = .006). Interestingly, two conditions are found to be associated with a lower risk of poorer mental health symptoms. They are “*I worry that economic instability will make it difficult to secure a stable career*” (*B* = 0.268, *p* = 0.028) and “*I feel anxious about how nuclear weapons or chemical warfare could create long-term environmental disasters*” (Stress *B* = 0.314, *p* < .001; Depression *B* = .367, *p* = .035).

**Table 2:** Odds ratio for the predictive role of threats and uncertainty towards risk of moderate to extremely severe mental health symptoms^n^ in young adults in Malaysia.

Threats and Uncertainty Question	Mod/High/Extreme Anxiety		Mod/High/Extreme Stress		Mod/High/Extreme Depression	
	*p*-value	Exp(*B*)	*p*-value	Exp(*B*)	*p*-value	Exp(*B*)
1. The thought of not having a job after graduation makes me stressed/anxious	.137	.170	.755	1.124	.798	1.149
2. Uncertainty about the future makes it difficult for me to plan my career path.	.573	2.139	.009	2.406*	.314	1.666
3. The pressure to meet family expectations makes me feel overwhelmed and emotionally drained.	.600	2.094	.941	.976	.143	.512
4. I sometimes sacrifice my psychological well-being to meet career and financial expectations.	.393	.397	.734	1.113	.487	.729
5. I worry that economic instability will make it difficult to secure a stable career.	.610	.487	.062	.440	.028	.268*
6. The rising cost of living makes me uncertain about achieving financial stability.	.629	.486	.069	.476	.929	1.058
7. Societal expectations make me feel obligated to prioritise financial success over personal fulfilment.	.846	.796	.595	.851	.885	1.070
8. Uncertainty about future economic conditions makes it difficult to save or invest for the long term.	.454	3.031	.320	1.417	.317	1.697
9. I feel pressure to achieve success quickly due to fears of future financial instability.	.184	.226	.580	1.206	.254	1.880
10. Financial instability causes me stress and anxiety about my future quality of life	.294	4.574	.013	2.517*	.209	2.001
11. Societal expectations about success and financial stability have an impact on my self-esteem and mental health.	.166	7.919	.678	1.145	.993	.995
12. The pressure to meet family expectations makes me feel overwhelmed and emotionally drained	.635	.512	.064	1.931	.006	5.706*
13. I worry that economic instability will limit my ability to maintain a good quality of life.	.946	1.106	.231	1.585	.616	1.317
14. I feel uncertain about starting a family due to concerns about financial and emotional stability.	.768	.703	.073	1.883	.480	1.435
15. I feel anxious about how nuclear weapons or chemical warfare could create long-term environmental disasters	.563	2.307	<.001	.314***	.035	.367*
16. Concerns about climate change negatively impact my mental well-being	.771	1.492	.192	.666	.751	1.163
17. I feel anxious about how environmental degradation will affect my future quality of life.	.507	2.864	.584	1.216	.326	.601
18. The unpredictability of climate-related disasters contributes to my stress and anxiety.	.946	.918	.997	.999	.524	1.352
19. The risk of war makes me feel uncertain about my personal safety and well-being.	.081	.178	.114	1.686	.589	1.280

n Based on moderate, high to extremely high scores of DASS-21 components.

*significant at *p*-value less than .05.

***significant at *p*-value less than .001.

Table 3 presents the findings for the association between threats and poorer psychological well-being (PWB) components, namely autonomy, environmental mastery, growth, positive relations, self-acceptance, and purpose of life. In summary, no significant associations were found between PWB autonomy and PWB self-acceptance, or between either and any of the items. Two items, “*The rising cost of living makes me uncertain about achieving financial stability*” (*B* = 0.485, *p* = .054) and “Uncertainty about future economic conditions makes it difficult to save or invest for the long term” (*B* = .557, *p* = .053), were found to predict lower odds of lower PWB environmental mastery scores. Meanwhile, “*Societal expectations make me feel obligated to prioritise financial success over personal fulfilment*” was found to be associated with a 1.7 times higher odds of lower PWB positive growth scores (*B* = 1.721, *p* = 0.028). In t1.7 times higher oddslation, worry related to financial “*Financial instability causes me stress and anxiety about my future quality of life*” (*B* = 2.283, *p* = 0.010) and concern related to war “*I feel anxious about how nuclear weapons or chemical warfare could create long-term environmental disasters*” associated with 1.8 times (*B* = 1.877, *p* = .057) greater odds of poorer score in this dimension. Interestingly, concern over unemployment (“*The thought of not having a job after graduation makes me stressed/anxious*”) was associated with lower odds of higher PWB positive relation scores (*B* = .446, *p* = .033). Additionally, concern over war, “*I feel anxious about how nuclear weapons or chemical warfare could create long-term environmental disasters*” (*B* = 2.848, *p* = .005), was also found to be associated with a 2.4 times increase in lower PWB purpose of life scores. Despite the result, “*I feel uncertain about starting a family due to concerns about financial*” item found to be associated with reduced odds of lower PWB purpose of life (*B* = .460, *p* = .019). In conclusion, the overall result from Tables 1–3 suggests a mixed combination of effects towards certain threat conditions towards the condition of QOL, mental health and PWB state of young adults. While some threats are associated with increased odds or risk of occurrence, others are associated with reduced odds, further suggesting a complex interplay among the findings.

**Table 3:** Odds ratio for the predictive role of threats and uncertainty towards risk of moderate to very poor PWB^n^ scores^,^ based on its six components among young adults in Malaysia.

Threats and Uncertainty Question	PWB autonomy		PWB environmental mastery		PWB positive growth		PWB positive relation		PWB self-acceptance		PWB purpose life	
	*p*-value	Exp(*B*)	*p*-value	Exp(*B*)	*p*-value	Exp(*B*)	*p*-value	Exp(*B*)	*p*-value	Exp(*B*)	*p*-value	Exp(*B*)
1. The thought of not having a job after graduation makes me stressed/anxious	.510	.825	.471	.800	.667	.882	.033	.446*	.469	1.241	.691	.875
2. Uncertainty about the future makes it difficult for me to plan my career path.	.220	1.360	.629	1.136	.133	.684	.638	.867	.980	.993	.818	.935
3. Family expectations add pressure to making financially secure career choices, even if they do not align with my interests.	.684	.898	.398	1.266	.667	.891	.831	.937	.941	.980	.579	1.186
4. I sometimes sacrifice my psychological well-being to meet career and financial expectations.	.366	1.253	.347	1.278	.491	.841	.290	.721	.120	.659	.536	.827
5. I worry that economic instability will make it difficult to secure a stable career.	.199	1.570	.467	1.317	.662	.853	.168	1.808	.995	1.002	.198	1.657
6. The rising cost of living makes me uncertain about achieving financial stability.	.220	.661	.054	.485*	.188	.645	.384	.698	.072	.529	.231	1.547
7. Societal expectations make me feel obligated to prioritise financial success over personal fulfilment.	.209	1.365	.929	1.024	.028	1.721*	.274	1.375	.097	1.529	.300	1.353
8. Uncertainty about future economic conditions makes it difficult to save or invest for the long term.	.604	.867	.053	.557*	.410	.797	.457	.778	.632	.871	.060	.531
9. I feel pressure to achieve success quickly due to fears of future financial instability.	.407	1.249	.090	1.616	.752	1.089	.381	.754	.151	1.489	.159	1.559
10. Financial instability causes me stress and anxiety about my future quality of life	.855	.951	.103	.616	.775	.924	.010	2.283*	.074	.589	.739	1.113
11. Societal expectations about success and financial stability have an impact on my self-esteem and mental health.	.572	1.162	.157	1.481	.927	.976	.311	.723	.274	1.351	.831	.935
12. The pressure to meet family expectations makes me feel overwhelmed and emotionally drained	.499	.824	.256	.706	.173	1.473	.776	.909	.820	1.070	.099	.565
13. I worry that economic instability will limit my ability to maintain a good quality of life.	.668	.882	.115	1.623	.468	1.237	.278	.676	.383	1.302	.263	.679
14. I feel uncertain about starting a family due to concerns about financial and emotional stability.	.510	.837	.095	.616	.109	.649	.054	.523	.895	1.038	.019	.460*
15. I feel anxious about how nuclear weapons or chemical warfare could create long-term environmental disasters	.406	.788	.660	.876	.076	.605	.057	1.877*	.573	.845	.005	2.484*
16. Concerns about climate change negatively impact my mental well-being	.993	.998	.916	.971	.918	.974	.550	.834	.206	.709	.653	.870
17. I feel anxious about how environmental degradation will affect my future quality of life.	.358	.762	.299	.722	.129	.641	.518	.793	.209	.677	.566	1.217
18. The unpredictability of climate-related disasters contributes to my stress and anxiety.	.745	.916	.653	1.136	.290	1.324	.308	.725	.349	1.299	.473	.796
19. The risk of war makes me feel uncertain about my personal safety and well-being.	.116	.651	.490	.820	.197	.707	.532	.819	.349	.768	.659	1.141

n Based on moderate to low levels of PWB components.

*significant at *p*-value less than .05.

## Discussion

The present study provides an extensive examination of how different types of threats and uncertainties contribute to variations in quality of life, mental health symptoms, and psychological well-being among young adults in Malaysia. Drawing on Bronfenbrenner's Ecological Systems Theory and the Threat Hypothesis, the findings reveal a complex interplay of effects, where certain threat conditions significantly increase risks to well-being. At the same time, others demonstrate weak or even protective associations. Overall, these results suggest that the degree of ecological proximity, perceived control, and relevance of each threat strongly influence how young adults respond to each scenarios, especially in the context of young adults in Malaysia.

### Predictive effects of threats towards poor quality of life (QOL)

Across QOL components, threats linked to economic instability consistently emerged as significant predictors of poor well-being outcomes. This pattern is consistent with the presumption of economic threats within the exosystem and mesosystem, where career planning and financial security are important considerations for many young people. Parallel to the threat hypothesis, threats perceived as immediate and personally relevant have a greater psychological impact, as this study's findings support. For example, concerns over unstable career prospects significantly predicted poorer physical and material well-being. Meanwhile, concerns about unemployment and the sacrifice of psychological well-being for career goals were associated with reduced relational quality of life. Such findings aligned with empirical findings concerning career anxiety among young adults (26). It is presumed that such threats directly interfere with everyday functioning, prompting heightened stress responses. Gellish et al. (31) suggested that intolerance to uncertainty influences young adults' psychological well-being. In contrast, most environmental, climate, and global political threats were not significant in these domains, likely because they are perceived as distant, less controllable, or less directly tied to daily responsibilities.

It is also very interesting to discover both the risk and protective effects of social, community, and civic domains of QOL in this study. Specifically, financial and family pressures significantly increased the likelihood of poorer outcomes among Malaysian young adults. Nevertheless, societal expectations around financial success appeared to reduce this risk. One possible explanation is that specific societal pressures become internalised as motivation, providing a sense of direction or structure. This is mentioned by Dai (27), who argues that social expectations drive good behaviour. From an ecological perspective, such expectations originate in the macrosystem but influence behaviour through values, norms, and identity development, as noted by Pastor et al. (28). This finding also suggests that not all external pressures are interpreted negatively. Notably, social expectations, norms and values can be promoted to navigate young adult behaviour through embedded personal aspirations or culturally endorsed life goals.

The personal development QOL domain had the most significant predictors in this study, including threats related to unemployment, financial uncertainty, family pressure, rising living costs, and even geopolitical instability. These threats disrupt long-term planning, goal setting, and self-exploration, all of which are key developmental tasks during emerging adulthood. The magnitude of these associations reinforces the idea that young adults are susceptible to uncertainties that impair their ability to envision or prepare for the future. This study also found that concerns about war and personal safety were associated with a 2.1-fold increase in the odds of poorer QOL related to personal development. War, from a social perspective, brings massive fatality and interruption to personal life. Aligned with many empirical and narrative studies, war disrupts economic relations, causing displacement and trauma across different levels (38). Other effects of war include the environment (Tucker & McNeil, 39) and food security (Behnassi & El Haiba, 40), both of which are closely connected to one's capacity to grow as a human. This indicates that young adults in Malaysia are concerned about their ability to develop, given the adversities of war across different chains of the system.

Many of the non-significant items within this domain were global or environmental threats that may feel less immediate or actionable, which could explain their weaker influence, especially considering the situational context of Malaysia, which faces less severe environmental threats when compared to the neighbouring countries, such as Indonesia (volcano eruption), the Philippines (typhoon), among others. Interestingly, recreation-related QOL was associated with only two significant threats, similarly centred around economic uncertainty and family pressure. The majority of threat items did not predict poorer recreational outcomes, suggesting that leisure activities are primarily influenced by tangible constraints such as time, energy, and resources rather than distant global threats.

### Predictive effects of threats towards mental health (MH) symptoms

The analysis of mental health symptoms revealed fewer significant predictors than the QOL outcomes, although the pattern remained consistent with ecological proximity. Career planning uncertainty and financial instability significantly increased the likelihood of anxiety and stress symptoms, while family-related pressures predicted higher levels of depressive symptoms. Specifically, financial pressure and pressure to succeed predict stress levels 2.4–2.5 times higher. Many global and environmental threats were not significantly related to MH symptoms. Presumably, this is due to habituation or to young adults differentiating between threats based on the degree of control. Family expectation was found to be the highest risk for depression symptoms, with 5.7 times higher, indicating the primary source of distress for young adult MH. This is not unique, given that Asian parents have high expectations for excellence and success as part of social norms. Such a finding is aligned with Rusli et al. (29) and Mossakowski (30), whereby the expectation set by the family on success is seen as one of the factors associated with symptoms of depression.

Unexpectedly, certain threat conditions showed protective effects, such as lower depression among those concerned about economic instability and reduced stress among those anxious about nuclear or chemical warfare. These counterintuitive findings may reflect adaptive coping processes and resilience mechanisms in which individuals appraise extreme global threats as separate from their personal circumstances to reduce internalised distress. It is also possible that concerns over shared uncertainties encourage stronger social bonding and support among peers, which may serve as a protective factor against poorer mental health outcomes. The opposite finding also warrants more investigation to understand young people's perception in Malaysia of peace-related threats, especially on any confounding effect, such as the Malaysian government's active participation as one of the regional leaders in leading peace-related discussions (i.e., Global Sumud flotilla) and efforts in the conflict region that indirectly shape such an optimistic stance. Another possible explanation is that such threats are perceived as geographically distant or less immediate, resulting in a degree of psychological distancing from the threat itself. Nevertheless, these interpretations remain speculative and warrant further investigation. Future studies should therefore focus on understanding the specific psychological processes underlying these findings, including how young adults perceive, interpret, and respond to broader socio-environmental threats, as well as the possibility of a psychological desensitisation effect given prolonged exposure to war content and global struggle in peace-related conflict.

### Predictive effects of threats towards poor psychological well-being (PWB)

The PWB results showed many non-significant findings, suggesting that broader aspects of well-being, such as autonomy, self-acceptance, and purpose in life, may be more stable and less easily disrupted by external threats in Malaysia. Nevertheless, societal expectations and financial instability were found to undermine specific components, such as growth and positive relations, with higher odds of 1.7 times and 2.3 times, respectively. This demonstrates that pressures among young adults may be rooted in a close ecological system and internalised as part of psychological burden. For example, concern over financial distress predicts a 2.3 times greater risk of a poor positive relation, suggesting the importance of this component as an indicator of good relations among young adults. This can be interpreted as a relationship with family and relatives, as well as marriage, both of which require financial confidence. A notable finding was that worry about unemployment was associated with stronger positive relations, possibly indicating that shared concerns encourage peer bonding and supportive interactions during times of uncertainty.

## Contribution and implication

The findings also provide several practical implications for policymakers and university support services. Given the significant role of career uncertainty, financial concerns, and family expectations in predicting poorer well-being outcomes, universities should strengthen career guidance programmes, financial literacy initiatives, and accessible mental health support services for students. University counsellors may also play an important role in identifying students experiencing distress related to these challenges, while providing early psychological support and resilience-building interventions. Such efforts are increasingly important amid growing uncertainty surrounding economic conditions, employment prospects, planetary sustainability, and global peace. These findings are particularly relevant in the context of the often silent and under-recognised psychological struggles experienced by many young adults as they navigate uncertainty about their future.

Creating more opportunities for young people to openly discuss and reflect on these challenges may help foster resilience, adaptive coping, and a more proactive outlook towards an increasingly uncertain global environment. Such initiatives may also encourage greater awareness of how broader socio-environmental conditions influence well-being, enabling young adults to better prepare for future challenges. At the policy level, efforts aimed at improving youth employment opportunities, financial security, and mental health awareness may help mitigate some of the socio-environmental stressors identified in this study. As concerns surrounding mental health continue to grow among young people in Malaysia, proactive interventions that address both psychological well-being and the broader social conditions contributing to distress are needed to prevent these challenges from developing into a more serious public health concern.

## Limitation and suggestion

Despite these findings, several limitations should be acknowledged. First, the regression models did not include demographic or socioeconomic covariates, as the primary objective of this study was to examine the predictive role of socio-environmental threat perceptions on well-being outcomes. Consequently, the possibility of residual confounding cannot be entirely ruled out. Future studies should incorporate relevant demographic and socioeconomic variables as control factors to further validate and strengthen the observed associations. Second, the present study included a relatively large number of predictors to provide a comprehensive assessment of socio-environmental threats. While this exploratory approach offers a broad understanding of potential risk factors, it may increase model variability and reduce stability. Future studies should validate the findings through cross-validation, replication, or independent samples to assess the robustness and generalisability of the observed associations. Third, the use of a binary response format may have simplified the complexity of threat perception by focusing only on the presence or absence of concern. Future research could adopt intensity- or frequency-based measures to better understand the varying degrees of threat experienced by young adults and their influence on well-being outcomes, thereby providing a more comprehensive understanding. Fourth, it is important to acknowledge that Malaysia is a diverse, multicultural society with varied ethnic, cultural, and socioeconomic backgrounds. The perception and impact of socio-environmental threats may differ across these groups due to differences in values, resources, lived experiences, and social support systems. Future studies should explore these differences more explicitly to provide a more nuanced understanding of young adult well-being in the Malaysian context.

## Conclusion

Taken together, the results demonstrate that threats exert differential influence depending on where they originate within the ecological system and how directly they intersect with young adults’ component of well-being. Threats that are immediate, personally relevant, and tied to socioeconomic or family expectations consistently increased the risk of poorer outcomes. Conversely, broader environmental and global threats often had no significant effect or were associated with unexpected protective patterns. These findings highlight the importance of understanding how young adults interpret, prioritise, and respond to different categories of threat. Strengthening financial preparedness, improving career support, and addressing family pressures may offer meaningful pathways for enhancing well-being while also acknowledging the adaptive strategies young adults employ to navigate an increasingly complex and uncertain world.

## Data Availability

The raw data supporting the conclusions of this article will be made available by the authors, without undue reservation.
